# Akt and phospholipase Cγ are involved in the regulation of growth and migration of MDA-MB-468 breast cancer and SW480 colon cancer cells when cultured with diabetogenic levels of glucose and insulin

**DOI:** 10.1186/1756-0500-5-214

**Published:** 2012-07-10

**Authors:** Nicola M Tomas, Kai Masur, Jonas C Piecha, Bernd Niggemann, Kurt S Zänker

**Affiliations:** 1 Institute of Immunology and Experimental Oncology, Witten/Herdecke University, Stockumer Str. 10, D-58448, Witten, Germany; 2 Institute for Plasma Research and Technology e.V. – INP Greifswald, Felix-Hausdorff-Str. 2, D-17489, Greifswald, Germany

**Keywords:** Breast and colon cancer, Diabetes mellitus type 2, Glucose, Insulin, Akt, PLCγ

## Abstract

**Background:**

Epidemiological studies revealed a strong correlation between the metabolic syndrome/diabetes mellitus type 2 (DM2) and higher incidence and faster progression of breast and colon cancer. However, the underlying molecular mechanisms are widely unknown. Akt and phospholipase Cγ (PLCγ) are involved in tyrosine kinase signaling and promote tumor cell growth and migration. Therefore, we examined regulatory functions and expression of Akt and PLCγ in a simplified in vitro diabetogenic model.

**Findings:**

Protein expression was determined by western blot analysis in MDA-MB-468 breast cancer and SW480 colon cancer cells previously cultured under physiologic (5.5 mM) and diabetogenic (11 mM) glucose concentrations (without and with 100 ng/ml insulin). We studied the culture effects on proliferation and migration of these cells, especially after inhibiting Akt and PLCγ. We found that Akt expression was up-regulated with high glucose and insulin in both cell lines, whereas PLCγ expression was enhanced in colon cancer cells only. High levels of glucose and insulin increased cell proliferation and migration in both cell lines in vitro, mediated by Akt and PLCγ, as shown through the specific pharmacological inhibitors A6730 and U73122.

**Conclusions:**

Our molecular data explain glucose- and insulin-induced changes in a cancer cell and help to understand what might trigger tumor cell proliferation and migration in DM2 patients, too.

## Background

Breast cancer is the most common type of cancer in women, and colon cancer is the third most common cancer in both sexes and the second leading cause of cancer deaths worldwide [1]. The majority of cancer patients, however, will not decease because of their primary tumor, but rather because of the spreading of metastasis, which is responsible for over 90% of cancer deaths [2].

The prevalence of obesity has increased dramatically over the last four decades, and it can be considered the leading health problem of the developed countries in the 21^st^ century. Obesity is strongly associated with the development of diabetes mellitus type 2 (DM2) and co-morbid diseases, such as hypertension and hyperlipidemia, commonly summarized as the metabolic syndrome [3].

Up-to-date retrospective and prospective epidemiological studies showed strong correlations between the metabolic syndrome and the incidence of malignant neoplasms in different organs [4,5]. Obesity increases the risk of breast and colon cancer [6,7]. DM2 is directly associated with a higher incidence and faster progression of several neoplasms including colon and breast cancer [5,8]. In particular, increased fasting serum insulin concentrations, commonly found in DM2 patients, seem to increase the risk for breast and colon cancer [9,10]. Besides, tyrosine kinases, namely the insulin receptor (IR) and insulin-like growth factor (IGF) receptors, are over-expressed in several human cancers, including cancer of the breast [11]. Consequently, over-expression of these receptors yields a selective growth advantage to breast cancer cells, especially in the presence of insulin resistance and associated hyperinsulinemia [12]. It has been known for over two decades that glucose is the driving force for tumor cell growth [13] and that high levels of insulin promote metastasis [14].

Insulin operates by binding to its target cell receptor, a heterotetrameric, transmembrane, multisubunit glycoprotein. The insulin signal is propagated through a phosphorylation network involving intracellular molecules such as phosphoinositid-3-kinase (PI3K), protein kinase B (PKB, Akt) and phospholipase Cγ (PLCγ). The serine/threonine kinase Akt is a well known regulator of widely divergent cellular processes, and it is implicated in intracellular insulin signaling. Akt has been described as an oncogene in several human cancers [15,16], and it is known to be a promoter of tumor cell proliferation [17], prolonged cell survival [18,19], and angiogenesis [20]. The phosphorylation and thus activation of Akt can be specifically inhibited by the trifluoroacetate salt hydrate A6730 [21]. Phospholipases (PLCs) are tyrosine kinase substrates that provide diacylglycerols (DAGs) for intracellular signaling in various contexts and its isoform PLCγ is known to be implicated in intracellular insulin signaling [22] and in colon and breast cancer progression [23,24]. U73122 is an aminosteroid that specifically inhibits PLCγ activation [25].

Given this knowledge about the gravity of glucose metabolism in tumor cells on the one hand and the growing number of studies investigating the epidemiological connection between the metabolic syndrome/DM2 and the development and progression of several human cancers on the other hand, surprisingly little is known about the underlying molecular changes and mechanisms that facilitate tumor development and progression when excessive glucose and insulin are available.

For these reasons, we report about the regulatory functions of Akt and PLCγ regarding tumor cell proliferation and migration under the specific premise of higher than normal glucose and insulin concentrations.

## Methods

### Cell culture

For all experiments the tumor cell lines SW480 colon adenocarcinoma and MDA-MB-468 mammary gland adenocarcinoma (both American Type Culture Collection, Manassas, VA, USA) were kept in RPMI 1640 media supplemented with 10% fetal bovine serum (PAA Laboratories, Pasching, Austria) and 1% penicillin/streptomycin (PAN Biotech GmbH, Aidenbach, Germany). The medium contained either 5.5 mM (99.1 mg/dl) glucose, 11 mM (198.2 mg/dl) glucose, or 11 mM glucose plus 100 ng/ml insulin. All cells were incubated at 37°C humidified atmosphere and 5% CO_2_ (Binder, Tuttlingen, Germany).

### Western blot analysis

The expression levels of Akt and PLCγ were analyzed by immunoblotting as described previously [26]. Cells (1 × 10^6^) were lysed in Laemmli sample buffer and incubated for 10 min at 95°C. Proteins were separated using SDS-PAGE and transferred to an PVDF-Immobilion-P membrane (Millipore, Schwalbach, Germany), followed by blocking with 10% milk powder (1 h at room temperature (RT)). After incubating the membrane overnight at 4°C with the primary monoclonal antibodies against Akt (Cell Signaling Technology, Boston, USA) and PLCγ (Sigma-Aldrich, Deisenhofen, Germany), membrane were washed vigorously with PBS-Tween. Subsequently, the membrane was incubated with appropriate anti-mouse or anti-goat peroxidase-linked secondary antibodies for 1 h at RT (all Southern Biotech, Birmingham, AL, USA), followed by incubation with the chemiluminescence blotting substrate (Roche Diagnostics, Mannheim, Germany) for 1 min at RT for visualization. Bands were detected using the Aequoria Macroscopic Imaging System (Hamatsu, Herrsching am Ammersee, Germany) and quantified using Wasabi 1.4 software (Hamatsu, Herrsching am Ammersee, Germany). The numbers shown are expressed as percentages of the 5.5 mM glucose control condition.

### Cell proliferation assay

Proliferation analysis was performed in a 96-well-plate with 10^4^ cells per well. Cells were incubated for 48 h at 37°C humidified atmosphere and 5% CO_2_ with and without the inhibitors A6730 and U73122 in a concentration of 500nM and 500 mg/dl, respectively (both Sigma-Aldrich, Deisenhofen, Germany). Then, according to the manual, 25μl of MTS color solution (Promega, Madison, WI, USA) was added directly to the culture wells and cells were then incubated under the same conditions for 2 h. Absorbance of the resulting colored formazan product was recorded at 490 nm in a 96-well-plate-reader. The quantity of formazan product is directly proportional to the number of living cells in culture.

### Cell migration assay

Cell migration was assessed using a three-dimensional collagen matrix assay followed by computer-assisted cell tracking [27]. A liquid collagen solution (purified collagen, Inamed Biomaterials, Fremont, CA, USA) was mixed with 10x MEM (Sigma, Taufkirchen, Germany), 7.5% sodium bicarbonate solution (Sigma, Taufkirchen, Germany), 10^5^ cells to be analyzed, and the inhibitors A6730 (500nM) and U73122 (500 ng/ml). Cell migration within the three-dimensional collagen matrix was recorded for 10 h at 37°C by time-lapse video-microscopy and subsequently analyzed by computer-assisted cell tracking. For analysis of the migratory activity, 30 cells per sample were randomly selected and paths were digitized as x/y coordinates in 15 min intervals. Migratory activity (MA) refers to the percentage of cells moving during a particular 15 min interval, and average migratory activity (AMA) is the average percentage of moving cells, calculated from all 15 min intervals in a 10 h period. Thereby, 100% MA was reached when all 30 cells migrated during the corresponding 15 min interval and 100% AMA was reached when all 30 cells migrated constantly over 10 h. The average distance migrated (ADM) in μm was calculated from 10 h of analysis.

### Statistical analysis

Data is presented as mean and standard deviation. Statistical significance was determined by Student’s *t*-test, whereby *p* < 0.05 was considered significant. All experiments were performed 3–6 times.

## Findings

To analyze Akt/PLCγ expression, proliferation capacity, and cell migration we cultured different tumor cell lines (MDA-MB-468 breast and SW480 colon cancer cells) at physiological (5.5 mM, control) and diabetogenic (11 mM) glucose concentrations without and with 100 ng/ml insulin.

**Figure 1:**
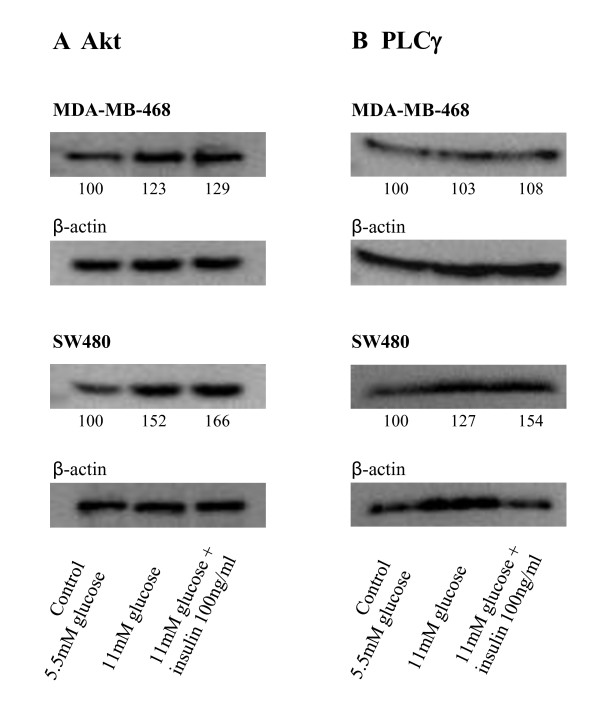
**Western blot analysis of Akt and PLCγ expression.** Western blot analysis was performed to evaluate Akt and PLCγ expression and shows that Akt expression was up-regulated in both cell lines when additional glucose was available (**A**). The combination of glucose and insulin caused an even higher up-regulation in both cell lines (**A**). PLCγ expression showed considerable increase in SW480 colon cancer cells exclusively (**B**). Numbers indicate relative Akt and PLCγ expression levels in relation to β-actin, whereby expression levels in the 5.5 mM glucose control media were set to 100%.

Raising the glucose concentration from 5.5 mM to 11 mM increased the expression of Akt by 23% in MDA-MB-468 breast cancer cells (Figure 1A). Addition of 100 ng/ml insulin to the 11 mM culture media increased Akt expression by another 6% when compared to cells cultured with 11 mM glucose alone. An even stronger effect could be seen for SW480 colon cancer cells, which raised Akt expression by 52% when cultured with high glucose concentrations. Here adding 100 ng/ml insulin caused a further increase of 14% (to 66%). The Akt-enhancing effect of insulin can be quantified as approximately one fourth of the glucose effect in both cell lines.

MDA-MB-468 cells did not significantly up-regulate phospholipase Cγ (PLCγ) expression neither when kept under higher than normal glucose concentrations alone, nor when 100 ng/ml insulin was added (Figure 1B). In SW480 cells high glucose and insulin levels showed an increase in PLCγ expression of 27% each, leading to a total increase of 54%.

**Figure 2:**
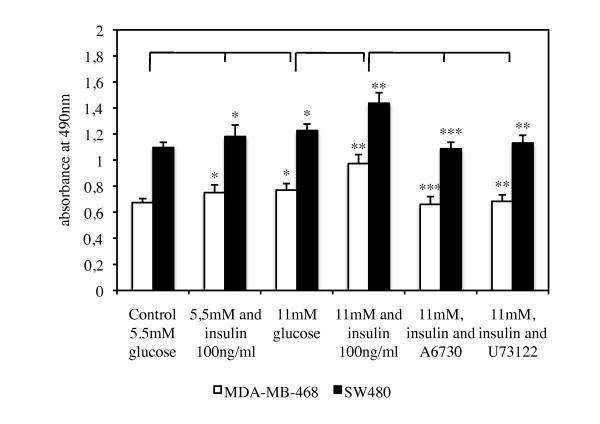
**Tumor cell proliferation with high glucose and insulin.** MDA-MB-468 breast cancer and SW480 colon cancer cells significantly increased proliferation rates after addition of insulin (100 ng/ml) and doubling glucose concentrations. Even higher proliferative capacity was seen when cells were simultaneously stimulated with glucose and insulin. SW480 cells showed consistently higher proliferation rates than MDA-MB-468 cells. Addition of AKT-inhibitor A6730 and PLCγ-inhibitor U73122 significantly reduced tumor cell proliferation to levels comparable with the control. *P*-values reflect 5.5 mM glucose plus insulin and 11 mM glucose against control, 11 mM glucose plus insulin against 11 mM glucose, and both culture conditions including the inhibitors against 11 mM glucose plus insulin. Thereby, *p <0.05; **p < 0.001; ***p < 0.0001.

We performed a proliferation assay in order to analyze the influence of both, high glucose and insulin concentrations, as well as inhibition of Akt and PLCγ on the proliferative capacity of the two tumor cell lines (Figure 2). In this assay, absorbance values at 490 nm are directly proportional to the number of living cells in culture. Both cell lines revealed significantly higher proliferation rates when cultured with additional insulin and hyperglycemic glucose concentrations. Thereby, cells stimulated with glucose (11 mM) had similar proliferation rates to cells stimulated with insulin alone (*p* = 0,2). Proliferation was highest when cells were simultaneously stimulated with glucose and insulin. Furthermore, inhibition of Akt and PLCγ by A6730 and U73122, respectively, reduced proliferative activity significantly when compared to glucose- and insulin-stimulated cell proliferation (Figure 2). On the other hand, no significant effect on tumor cell proliferation was seen after adding A6730 and U73122 to the control media (data not shown). SW480 cells displayed significantly higher proliferation rates than MDA-MB-468 cells when kept under normal and increased glucose and insulin concentrations (*p* < 0.001 for all three conditions).

**Figure 3:**
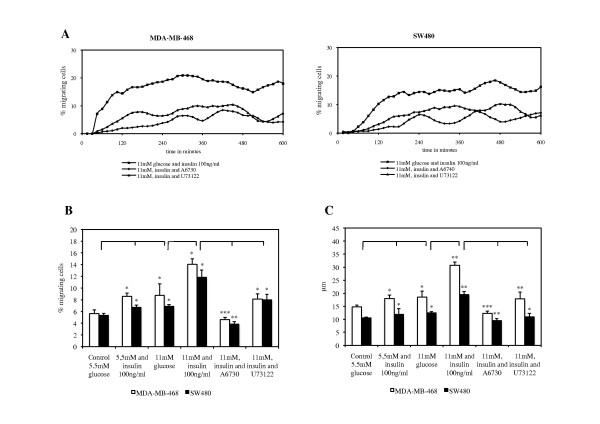
**Tumor cell migration with high glucose and insulin.** MDA-MB-468 breast cancer and SW480 colon cancer cells reduced migratory activity (MA) after addition of Akt-inhibitor A6730 and PLCγ-inhibitor U73122 when compared to cells stimulated with increased glucose and insulin (**A**). MA is the percentage of cells moving at a particular 15 min interval. Both cell lines significantly increased average migratory activity (AMA, **B**) and average distance migrated (ADM, **C**) after stimulation with glucose and insulin. AMA is the average percentage of moving cells, calculated from all 15 min intervals in a 10 h period. ADM refers to the average distance in μm that a tumor cell covered during 10 h of analysis. Akt-inhibitor A6730 and PLCγ-inhibitor U73122 significantly reduced AMA and ADM when compared to cells stimulated with increased glucose and insulin, emphasizing the regulatory functions of Akt and PLCγ when glucose and insulin concentrations are high. *P*-values reflect 5.5 mM glucose plus insulin and 11 mM glucose against control, 11 mM glucose plus insulin against 11 mM glucose, and both culture conditions including the inhibitors against 11 mM glucose plus insulin. Thereby, *p < 0.05; **p < 0.001; ***p < 0.0001.

High glucose and insulin concentrations significantly increased migratory activity (MA) in the investigated cell lines. MA refers to the percentage of cells moving during a partiular 15 min interval. Akt-inhibitor A6730 and PLCγ-inhibitor U73122 significantly reduced MA in both cell lines (Figure 3A). This glucose- and insulin-induced increase in cell migration is also reflected by an augmentation in average migratory activity (AMA), i.e. the average percentage of moving cells, calculated from all 15 min intervals in a 10 h period (Figure 3B). Maximum MA in MDA-MB-468 and SW480 cell lines was seen after concurrent stimulation with 11 mM glucose and 100 ng/ml insulin. It could be observed that both cell lines achieved this increase in MA by longer episodes of migration and shortening of pauses. Both inhibitors were able to annihilate the stimulating effect of insulin. Thereby, addition of U73122 resulted in migratory levels comparable to cells that were stimulated by a high level of glucose alone (11 mM) and thus only abolished the inducing effect of insulin. In comparison, A6730 reduced migration even below the control condition (5.5 mM) in both cell lines (Figure 3B).

The fact that glucose and insulin were strong inducers of tumor cell migration is also reflected by an increase in average distance migrated (ADM, Figure 3C). ADM refers to the average distance in μm that a cell covered during 10 h of analysis. Both, glucose and insulin, significantly elongated ADM, regardless of the cell type. Cells that were exposed to one of the inhibitors significantly reduced ADM when compared to cells cultured with 11 mM glucose and 100 ng/ml insulin. A6730 reduced ADM to a greater extent than U73122 in both cell lines (Figure 3C). Both inhibitors showed no significant effects on migratory parameters when added to the control media (data not shown).

Regarding the distance migrated over 10 h, MDA-MB-468 breast cancer cells appear to be more susceptible to glucose- and insulin-stimulation than SW480 colon cancer cells (*p* < 0.05 for 11 mM glucose, *p* < 0.001 for 11 mM glucose plus insulin). We observed that this effect was due to a greater augmentation in migratory velocity.

## Discussion

Tumor staging – including tumor size and invasiveness, lymphatic tissue involvement, and spreading to distant organ sites – is the main predictor of the prognosis for most solid organ tumor patients. Thus, finding biochemical signatures that define a tumor’s potential to metastasize can build the basis for new ways of determining a patient’s prognosis and eventually lead to new therapeutic targets. Glucose metabolism and its regulation, i.e. transcellular glucose transport and hormonal control via insulin and modification of the following signaling pathways, are such possible signatures. The results presented in this work strongly suggest that the glucose- and insulin-induced changes in proliferation and migration of MDA-MB-468 breast cancer and SW480 colon cancer cells are mediated by changes in Akt and/or PLCγ signaling. The investigated cell lines are widely used in cancer research and are well characterized in the context of their metabolic response to increased availability of glucose and insulin [28,29].

Akt is activated in most tumors [30] and various oncogenic functions, such as induction of migration [31] and metastasis [32], proliferation [17], and prolonged cell survival [18], have been described. Interestingly, Akt also enhances cellular glucose uptake by mobilizing glucose transporters to the cell surface [33]. In 2010, Johnson et al. [34] showed that proteins of the phosphoinositid-3-kinase (PI3K)/Akt pathway are significantly over-expressed in colorectal cancer compared to healthy cells, and that expression levels correlated with cancer stage. PI3K is activated by tyrosine kinase receptor stimulation, especially by the binding of insulin [35]. PI3K mediates the generation of phosphatidylinositol (3,4,5)-trisphosphate (PIP3) which allows Akt to be phosphorylated and thus activated [36]. The increased expression of Akt that we found with high glucose and insulin provides augmented substrate for the PI3K-pathway and may explain the increased proliferation and migration of the investigated cell lines when stimulated by glucose and insulin. The fact that addition of the Akt-specific inhibitor A6730 markedly decreased cell migration and proliferation with high glucose and insulin emphasizes that the up-regulation of Akt plays a crucial role in the processes of proliferation and migration. We could also show that the inhibitory effects of A6730 were specific to the setting with high glucose and insulin as no inhibitory effects of A6730 were evident when used without simultaneous stimulation by glucose and insulin.

In breast cancer patients, PLCγ was shown to be up-regulated in distant metastases when compared with the primary tumor, indicating its involvement in metastasis formation [23]. In our experiments, increased glucose and insulin caused an up-regulation of PLCγ in SW480 colon cancer cells, but no up-regulation in MDA-MB-468 breast cancer cells. However, migration and proliferation could be reduced significantly by adding U73122 to the diabetic culture conditions. This highlights the regulating capacity of PLCγ in both cell types. Thereby, the inhibitory effect of U73122 was specific to the condition with increased glucose and insulin as no decrease in proliferation and migration was seen when U73122 was used with normal glucose and no insulin.

How the treatment of diabetes is linked to the incidence of several cancers has gained a lot of interest over the last years and alarming data has recently been published. Hemkens *et. al.*[37] found that patients treated with human insulin or insulin analogues had a higher incidence of malignant neoplasms than patients who were not treated with insulin. Furthermore, the risk of developing cancer increased with higher dosages of insulin. In 2010, Baur et al. [38] stated that among diabetic patients, those treated with insulin had a four-fold higher risk to die from cancer. However, patients treated with the insulin-sensitizing antidiabetic agent metformin had mortality levels comparable with those of non-diabetic patients. Our in vitro results support these findings and help to explain how insulin and insulin therapy may increase cancer progression: from our point of view, insulin is a strong inducer of tumor cell migration and proliferation and a promoter of oncogenes such as Akt and PLCγ. This might indicate that, when patients show a family history for cancer, the use of long-acting insulin in the treatment of DM2 should be reconsidered.

## Conclusions

Our data show how glucose and insulin change proliferation and migration capacity in MDA-MB-468 breast cancer and SW480 colon cancer cells in vitro. Our results help to understand the diverse roles of glucose and insulin – energy supply and modulation of signaling cascades by modifying Akt and PLCγ expression – and thus explain which changes on a molecular, transcriptional level could be responsible for the epidemiological connection between the metabolic syndrome/DM2 and the progression of various malignancies. Considering our findings regarding the essential role of Akt in tumor progression, pharmacological research targeting this oncogene might be particularly promising.

## Abbreviations

ADM = average distance migrated; AMA = average migratory activity; DAG = diacylglycerol; DM2 = diabetes mellitus type 2; IR = insulin receptor; IGF = inslin-like growth factor; MA = migratory activity; PI3K = phosphoinositid-3-kinase; PIP3 = phosphatidylinositol (3,4,5)-trisphosphate; PKB = protein kinase B; PLC = phospholipase; PLCγ = phospholipase Cγ.

## Competing interests

The authors declare that they have no competing interests.

## Authors’ contributions

NMT performed the experiments, analyzed and interpreted the results, created the figures, and drafted the manuscript. KM and KSZ designed the study and revised the manuscript. JCP contributed to the experiments and revised the manuscript. BN supported the analysis of the cell migration data. All authors have read and approved the final manuscript.
